# The use of ECMO in pediatric granulomatosis with polyangiitis

**DOI:** 10.1186/s12969-022-00693-8

**Published:** 2022-05-10

**Authors:** Rachel Finkel, Jesse Honig, Chun P. Chao, Erin Rescoe, Sonia Solomon

**Affiliations:** 1 Department of Pediatrics, Maria Fareri Children’s Hospital, Westchester Medical Center, New York Medical College, Valhalla, New York USA; 2 Department of Pediatrics, New York Medical College, Valhalla, New York USA; 3 Division of Rheumatology, Maria Fareri Children’s Hospital, Westchester Medical Center, Boston Children’s Health Physicians, New York Medical College, Valhalla, New York USA; 4 Division of Critical Care Medicine, Maria Fareri Children’s Hospital, Westchester Medical Center, Boston Children’s Health Physicians, New York Medical College, Valhalla, New York USA; 5 Division of Pediatric Nephrology, Maria Fareri Children’s Hospital, Westchester Medical Center, Boston Children’s Health Physicians, New York Medical College, Valhalla, NY USA

**Keywords:** AAV, ANCA, ECMO, HD, Vasculitis

## Abstract

**Background:**

Granulomatosis with polyangiitis (GPA) vasculitis with pulmonary-renal syndrome rarely presents in children and is associated with high mortality rates.

**Case presentation:**

We present the case of a 13-year-old male with newly diagnosed GPA vasculitis, treated with extracorporeal membrane oxygenation, continuous renal replacement therapy, plasmapheresis, rituximab, cyclophosphamide, and corticosteroids.

**Conclusion:**

This case presentation demonstrates that ECMO can be used as a life supporting therapy in pediatric patients with pulmonary hemorrhage from ANCA vasculitis in conjunction with other therapies.

## Introduction

Antineutrophil cytoplasmic antibody (ANCA) associated vasculitis is a multisystemic disease process often resulting in necrotizing arteritis, with immune complex deposits observed in small to medium-sized vessels, including ANCAs directed against proteinase 3 (PR3) and myeloperoxidase (MPO) [1]. ANCA associated vasculitides are predominantly observed in adults, and rarely present in pediatric populations. The most frequently observed subtype of ANCA-associated vasculitides seen in children is granulomatosis with polyangiitis (GPA) vasculitis, which presents with a wide variety of symptoms. GPA vasculitis is classically associated with a triad of upper and lower respiratory tract involvement with renal involvement. While renal involvement is relatively common, seen in 65% of pediatric GPA cases [2], pulmonary-renal syndrome is a much rarer manifestation associated with high mortality rates, with diffuse alveolar hemorrhage observed in 5–10% of patients [3]. In GPA vasculitis, for those with severe refractory respiratory failure, particularly those due to diffuse alveolar hemorrhage, veno-venous extracorporeal membrane oxygenation (VV-ECMO) can be used as rescue therapy [4, 5]. VV-ECMO is life sustaining therapy for patients with refractory respiratory failure with reversible causes [4]. However, ECMO does not come without risks, which include bleeding, infection, and strokes [6]. Even with ECMO treatment, survival rates for ANCA-associated vasculitis vary, with the main data available being case reports [7]. In this case report, we describe the presentation, clinic course, and outcome of a pediatric patient with GPA requiring immunosuppression, renal replacement therapy, and successful VV-ECMO.

## Case description

A 13-year-old male with no past medical history presented to our emergency department (ED) with one day of fever (Tmax 103.3), three weeks of fatigue, myalgias, mild cough, two episodes of hemoptysis, and shortness of breath. In the ED, he was noted to have the following vital signs: temperature of 98.0° F, heart rate of 96 beats per minute, respiratory rate of 50 breaths per minute, pulse oximeter reading of 93% on room air, and blood pressure of 119/77. His respiratory exam was most notable for diffuse crackles which were worse at the bases of both lungs. His CBC demonstrated a white blood cell (WBC) count of 8.1 k/mm^3^, normocytic anemia (Hgb 9.7 g/dl, Hct 29%), and a platelet count of 261 k/mm^3^. Additional bloodwork demonstrated acute renal insufficiency with a blood urea nitrogen (BUN) level of 86 mg/dL and a creatinine (Cr) of 8.06 mg/dL. Electrolytes were normal apart from a mild metabolic acidosis, bicarbonate 17 mEq/L, and hyperkalemia with a potassium of 5.7 mEq/L. Complement levels were normal. C-reactive protein (CRP) was 18 mg/dL and erythrocyte sedimentation rate (ESR) was 55 mm/hr. His urine demonstrated microscopic hematuria (149 RBCs/high power field) and sub-nephrotic proteinuria (urine protein to creatinine ratio of 1 g/g). ANCA laboratory tests were sent in the ED given his constellation of symptoms. He was admitted to the pediatric intensive care unit due to respiratory insufficiency and acute renal failure. The initial exam performed by the admitting intensivist noted that the patient was tachypneic with shallow respirations and had decreased breath sounds on right. An initial chest x-ray demonstrated a dense right infiltrate. On admission, he was placed on continuous positive airway pressure (CPAP) for respiratory support. By hospital day two, he was noted to have improvement in his respiratory status and was weaned from CPAP to nasal cannula. Given the improvement in his respiratory status and the need to establish a diagnosis for his worsening renal insufficiency (BUN 88 mg/dL and Cr 9 mg/dL) the decision was made that the patient was stable enough to proceed with a renal biopsy. However, during induction of anesthesia, he developed frank pulmonary hemorrhage requiring intubation. The hemorrhage resolved with positive pressure ventilation. Computed tomography of his chest demonstrated concern for alveolar hemorrhage. At that time, laboratory results returned positive for c-ANCA antibodies (elevated PR-3) but were negative for p-ANCA. His other rheumatologic labs were found to be negative. He also was noted to have significant anemia secondary to pulmonary hemorrhage (Hgb 5.4, Hct 15.9%). Renal biopsy was thus deferred until his respiratory status stabilized and was completed at bedside on hospital day three. Given his pulmonary hemorrhage, completed renal biopsy, and laboratory findings consistent with GPA, he was started on plasmapheresis, pulse steroids with intravenous (IV) methylprednisolone and rituximab (375 mg/m^2^/dose) as part of induction therapy. His BUN/Cr continued to rise, reaching a maximum of BUN 113 mg/dl and Cr 10.09 mg/dL. He was able to make adequate urine, but due to worsening uremia, he was started on intermittent hemodialysis (HD). His respiratory status deteriorated due to recurrent hemorrhage. He failed conventional ventilation and was escalated to high frequency oscillatory ventilation (HFOV) in an effort to tamponade the bleeding. At that time, his renal biopsy results demonstrated pauci-immune crescentic glomerulonephritis, consistent with ANCA-mediated renal disease.

Unfortunately, he continued to have active pulmonary hemorrhage and developed pneumothoraces and pneumomediastinum from high mean airway pressures. He had poor oxygen saturations, saturating in the 60’s. He required multiple chest tubes for adequate lung re-expansion. Given his complications from high mean airway pressures and worsening hypoxemia from airway hemorrhage, and concern for impending cardiac arrest, it was decided to cannulate for VV- ECMO. The goal was to provide adequate oxygen delivery until blood could be cleared from his airways through therapeutic bronchoscopy. To minimize the risk of rebleeding, the ECMO circuit was not anticoagulated for 48 h after which time low-dose heparin was started. Once the patient was clinically stable, a bronchoscopy was able to remove significant blood clots in his airways which had been the cause of his refractory hypoxemia. While on VV-ECMO, daily intermittent HD was transitioned to continuous veno-venous hemodialysis (CVVHD) due to his critical status. In summary, his induction treatment included the following: methylprednisolone (a total of 3 doses of 30 mg/kg/dose, maximum of 1 g/dose), rituximab (375 mg/m^2^/weekly dose administered 4 times), plasmapheresis (a total of five rounds), and intravenous immunoglobulins (IVIG). Given that his clinical status continued to worsen, he was also given low dose cyclophosphamide (15 mg/kg) with mesna (225 mg, for bladder protection) for two doses, two weeks apart. He was decannulated after 16 days and extubated after 21 days later. He remained on CVVHD for a total of eight days and subsequently became anuric. He was transitioned to intermittent hemodialysis, which was discontinued after 40 days. Secondary to his renal insufficiency, he also developed hypertension, which was managed with nicardipine and esmolol intravenous drips and later transitioned to oral amlodipine, atenolol, and clonidine for subsequent management.

Considering his significant risk of superimposed infection given persistent fevers and leukocytosis, his prednisone was ultimately weaned to 20 mg daily. Upon discharge, his hemoglobin was 12.5 g/dL, Hct 37.5%, his serum creatinine was 1.97 mg/dl with mild uremia (BUN 61 mg/dL), and his other electrolytes were within normal range. He was making approximately 2 L of urine per day (1.6 mL/kg/hr).

Four weeks after discharge, his BUN and creatinine began to rise again (100 mg/dL and 3.8 mg/dl, respectively). In hopes of recovering his renal function even further, he was started on prednisone 60 mg daily and azathioprine 50 mg daily (renally dosed). Azathioprine was initiated with hopes of preserving his renal function. Despite intensifying his immunosuppression, his serum Cr slowly increased from 3.8 mg/dl to 6.7 mg/dl within another six weeks. His BUN also continued to rise (maximum value of 110 mg/dl). In the setting of worsening renal function despite of intensifying immunosuppression, hemodialysis was initiated and later transitioned to peritoneal dialysis. After starting dialysis, his azathioprine and steroids were then weaned off and he was maintained on rituximab approximately every 6 months. After one year of disease quiescence, he successfully received a kidney transplant.

## Discussion

ANCA vasculitis is a rare disease, especially in the pediatric setting. Most case reports describing the use of ECMO in ANCA vasculitis focus on the adult population [7–10]. There are very few case reports, especially within the pediatric population, described in the past 10 years detailing a patient’s hospital course with successful use of ECMO [11]. This is the third report of its kind describing a pediatric patient whose treatment involved multiple immunosuppressive medications and ECMO promoting his survival from a disease with a high mortality [11, 12].

In a case series of eight patients on ECMO from various causes of pulmonary hemorrhage (i.e., GPA, sepsis, systemic lupus erythematosus, and autoimmune hepatitis), two patients had GPA and were also successfully treated with ECMO, plasmapheresis, systemic steroids, and cyclophosphamide. One patient was treated with veno-arterial ECMO (VA-ECMO) and the other seven with VV-ECMO [13]. Another case report describes a similar patient scenario of a 13-year-old male with MPA, requiring VA-ECMO and continuous renal replacement therapy, in addition to plasmapheresis, corticosteroids, cyclophosphamide, and rituximab [11], which is similar to the treatment received by our patient.

These case studies suggest that patients with ANCA vasculitis who also have pulmonary hemorrhage may benefit from additional respiratory support while waiting for the full effects of inductive immunosuppressive agents. Some clinicians have found success utilizing ECMO specifically in the management of diffuse alveolar/pulmonary hemorrhage secondary to ANCA vasculitis [8–10]. Utilizing ECMO provided the support our patient needed to survive while awaiting the improvement in arteritis, which was the underlying etiology for the pulmonary hemorrhage and ensuing respiratory failure. This support also allowed for a therapeutic bronchoscopy to be performed safely. The bronchoscopy enabled the removal of significant blood clots from his airways, which was necessary for recovery of native lung function. It is also interesting to note that in the case series with eight patients mentioned above [13], seven out of the eight patients were treated with VV-ECMO and not VA-ECMO. This is significant because VV-ECMO is easier to manage without systemic anticoagulation, which is clearly a benefit in patients, such as ours, with active bleeding. The same case series also did not report any bleeding complications. However, even in one case report involving a patient with active pulmonary hemorrhage where initially no systemic coagulation was added but subsequently involved heparinizing the circuits due to active clots being formed, resolution of bleeding occurred by day three of ECMO, which suggests that ECMO can be safe in smaller children with active pulmonary hemorrhage as well [9]. In addition, VV-ECMO carries less risks than VA-ECMO, such as stroke. The patient was on ECMO for a total of 16 days, like previous patients described, survived decannulation, extubation, and was discharged home. In our case, we were able to use adult sized cannulas and circuits, which minimized the need for anticoagulation and mitigated the risk for continued pulmonary hemorrhage. Although the cases above describe success with ECMO therapy, ECMO remains a rescue therapy for both pediatric and adult patients with pulmonary hemorrhage due to the concern for bleeding complications associated with ECMO itself, as well as the risk of infection in immunocompromised patients. Prospective studies are needed to quantify the risk in our specific pediatric population.

Immunosuppressive treatment for ANCA vasculitis mainly involves two stages: induction therapy for the first 3–6 months to induce remission followed by maintenance therapy for the next 2–4 years with the aim of preventing relapses [14]. For patients with ANCA vasculitis with rapidly progressive glomerulonephritis or alveolar hemorrhage, pulse steroid treatment with IV methylprednisolone is often used for three days, as done with our patient. Previously, the standard of care for induction therapy involved glucocorticoids in combination with cyclophosphamide. However, given the toxicity profile of cyclophosphamide, rituximab has been studied as an alternative agent in adults and is increasingly being utilized and preferred (dose: 375 mg/m^2^ of body surface area per week for 4 weeks) [9, 15]. In particular, two important randomized control trials have been conducted: the RAVE and RITUXIVAS trials. The RAVE trial enrolled patients with new and relapsing ANCA vasculitis [16]. One arm received cyclophosphamide and the other received rituximab [16]. The end point was tapering off steroids by 6 months. Rituximab was found to be non-inferior to cyclophosphamide in reaching this goal. However, this study did not include patients requiring renal replacement therapy [16]. The RITUXIVAS trial only included patients with newly diagnosed ANCA vasculitis and included those with renal involvement [17]. Patients received either glucocorticoids plus rituximab with two IV doses of cyclophosphamide or IV cyclophosphamide for three to six months followed by azathioprine [17]. The end points were sustained remission rates at 12 months and severe adverse events [17]. Once again, rituximab was not found to be inferior [17]. Thus, rituximab is now often preferred in patients with relapsing disease, refractory disease, and contraindications to cyclophosphamide [15]. In addition to rituximab, there are several other induction treatment options being examined such as mycophenolate mofetil [18], combining cyclophosphamide and rituximab [19], and utilizing complement inhibitors [20]. As described above, our patient received rituximab in addition to glucocorticoids as part of his induction therapy. Although the PEXIVAS study demonstrated that plasmapheresis has not been shown to reduce the incidence of death or end stage renal disease, given our patient’s acute decompensation, the decision was made to proceed with plasmapheresis [21]. Plasmapheresis, cyclophosphamide, and IVIG were subsequently added given his ongoing clinical decline, as others have done in severe renal/life-threatening disease.

Cyclophosphamide used to be the cornerstone of maintenance therapy as well, with the goal of maintenance therapy being to prevent relapse rates. However, again, other options have been evaluated given the toxicity of cyclophosphamide. Given that we initially started with IV methylprednisolone, rituximab, and plasmapheresis, we only chose to use cyclophosphamide after our patient continued to further decompensate and required ECMO. Currently, azathioprine and/or rituximab are preferred forms of treatment [15]. The MAINRITSAN trial found that in patients who achieved remission after induction therapy with rituximab, when given rituximab infusions every six months patients had less major relapse at month 28 compared to the group of patients who only received azathioprine [22].

In pediatric cases of ANCA vasculitis, much of the current treatment regimens are extrapolated from adults as there are no pediatric randomized control trials evaluating treatment for pediatric ANCA vasculitis. Given the paucity of data in pediatrics, there are guidelines from various organizations. The SHARE (Single Hub and Access point for Pediatric Rheumatology in Europe) initiative, which has provided the only pediatric specific guideline, recently developed clinical care guidelines for the management of pediatric vasculitis [23]. Guidelines for induction focus on corticosteroids and cyclophosphamide as the main agents, as was common in adults. Rituximab, unlike in adults, was not discussed to be a first line induction agent but rather as second or third line. Plasma exchange therapy, while not commonly used in adult patients, was considered as an induction agent for pediatric patients if primary induction agents fail [14]. Although rituximab is thus far not mentioned as first line agent in induction or maintenance therapy, it is used more frequently in treating ANCA vasculitis [23].

This case report demonstrates the aggressive nature of ANCA vasculitis. Although this is a rare disease, modern immunosuppressive agents have been successfully used. Most importantly, our case report demonstrates and corroborates similar findings to those of other reports, which demonstrate that ECMO can be used as a life supporting therapy in pediatric patients with pulmonary hemorrhage from ANCA vasculitis. We hope that the information presented here about our patient may be useful to other patients and providers.

## Data Availability

N/A.

## Abbreviations

ANCA: Antineutrophil cytoplasmic antibody
PR3: Proteinase 3
MPO: Myeloperoxidase
GPA: Granulomatosis with polyangiitis
ECMO: Extracorporeal membrane oxygenation
VV-ECMO: Veno-venous extracorporeal membrane oxygenation
VA-ECMO: Veno-arterial extracorporeal membrane oxygenation
ED: Emergency department
CBC: Complete blood counte
WBC: White blood cell
BUN: Blood urea nitrogen
Cr: Creatinine
CRP: C-reactive protein
ESR: Erythrocyte sedimentation rate
CPAP: Continuous positive airway pressure
HD: Hemodialysis
HFOV: High frequency oscillatory ventilation
CVVHD: Continuous veno-venous hemodialysis
IVIG: Intravenous immunoglobulins
